# Rapid alteration of protein phosphorylation during postmortem: implication in the study of protein phosphorylation

**DOI:** 10.1038/srep15709

**Published:** 2015-10-29

**Authors:** Yifan Wang, Yanchong Zhang, Wen Hu, Shutao Xie, Cheng-Xin Gong, Khalid Iqbal, Fei Liu

**Affiliations:** 1 Department of Neurochemistry, Inge Grundke-Iqbal Research Floor, New York State Institute for Basic Research in Developmental Disabilities, Staten Island, New York 10314, USA; 2 Jiangsu Key Laboratory of Neuroregeneration, Co-innovation Center of Neuroregeneration, Nantong University, Nantong, Jiangsu 226001, P. R. China; 3 School of Pharmacy, Nantong University, Nantong, Jiangsu 226001, P. R. China

## Abstract

Protein phosphorylation is an important post-translational modification of proteins. Postmortem tissues are widely being utilized in the biomedical studies, but the effects of postmortem on protein phosphorylation have not been received enough attention. In the present study, we found here that most proteins in mouse brain, heart, liver, and kidney were rapidly dephosphorylated to various degrees during 20 sec to 10 min postmortem. Phosphorylation of tau at Thr212 and glycogen synthase kinase 3β (GSK-3β) at Ser9 was reduced by 50% in the brain with 40 sec postmortem, a regular time for tissue processing. During postmortem, phosphorylation of cAMP-dependent protein kinase (PKA) and AMP activated kinase (AMPK) was increased in the brain, but not in other organs. Perfusion of the brain with cold or room temperature phosphate-buffered saline (PBS) also caused significant alteration of protein phosphorylation. Cooling down and maintaining mouse brains in the ice-cold buffer prevented the alteration effectively. This study suggests that phosphorylation of proteins is rapidly changed during postmortem. Thus, immediate processing of tissues followed by cooling down in ice-cold buffer is vitally important and perfusion has to be avoided when protein phosphorylation is to be studied.

Protein phosphorylation was first reported in 1906 by Phoebus Levene^1^. However, phosphorylation as a regulatory physiological mechanism was discovered by Eddie Fischer and Ed Krebs in 1955^2^. Phosphorylation can modify a protein function by many ways, such as activating or inactivating its biological activity, stabilizing or marking it for degradation, affecting its subcellular localization and initiating or disrupting protein–protein interactions. The reversible phosphorylation of proteins regulates nearly every aspect of cell life^3^, which includes regulation of signaling pathways and cellular processes that mediate metabolism, transcription, translation, cell-cycle progression, differentiation, cytoskeleton arrangement and cell movement, apoptosis, intercellular communication, and neuronal and immunological functions^4^. It is believed that perhaps 1/3 of the proteins encoded by the human genome are phosphoproteins with tens of thousands of distinct phosphorylation sites^5^. Importantly, abnormal phosphorylation is now believed as a cause or consequence of many human diseases including Alzheimer’s disease^5^,^6^,^7^.

Protein phosphorylation is a post-translational modification of proteins in which a serine, threonine or tyrosine residue is phosphorylated by a protein kinase by the addition of a covalently bound phosphate group. Its reverse reaction, called dephosphorylation, is catalyzed by protein phosphatases. Protein kinases and phosphatases work independently and in a balanced manner to regulate the state of phosphorylation and thereby the function of proteins. The human genome contains about 500 protein kinase genes and they constitute about 2% of all human genes^8^. However, there are much less number of protein phosphatases, and protein phosphatases have much broader substrate specificities than protein kinases.

Microtubule associated protein tau is a major neuronal microtubule associated protein. It is hyperphosphorylated in the brain of individuals with Alzheimer’s disease (AD)^6^,^7^. More than 40 phosphorylation sites have been identified in tau from AD brain^9^. We recently found that tau protein was rapidly dephosphorylated in mouse brain after death, which triggered us to study the progress of protein dephosphorylation during postmortem interval. We found that in addition to tau, most proteins are rapidly dephosphorylated after death in a site-, protein-, and tissue specific manner. Dephosphorylation of some proteins after death progresses in seconds. Instantly cooling down the tissues in the ice-cold buffer following death prevents protein dephosphorylation.

## Results

### Rapid dephosphorylation of tau at multiple phosphorylation sites in the mouse brain during postmortem

**Figure 1:**
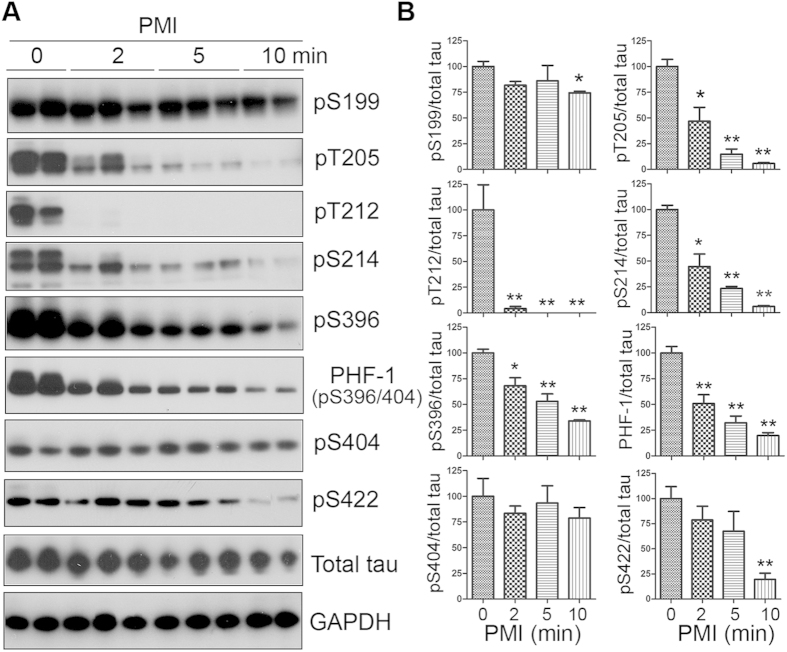
Tau is rapidly dephosphorylated site-specifically in mouse brains during postmortem. Mice were scarified by cervical dislocation. The dead animal bodies were kept at room temperature for the indicated periods of time. (**A**) Phosphorylation of tau was analyzed by Western blots developed with phosphorylation-dependent and site-specific tau antibodies indicated at the right side of the blots. (**B**) The levels of tau phosphorylation at individual sites were quantified and normalized with total tau level and presented as mean ± SEM. (n = 3–4). *p < 0.05; **p < 0.01.

Tau is phosphorylated and dephosphorylated by multiple kinases and phosphatases in site specific manner, respectively. To study the changes in tau phosphorylation after death in mouse brains. We sacrificed the mice by cervical dislocation and left the dead bodies at room temperature for 2, 5, and 10 min. The forebrains were collected and stored at −80 °C. Phosphorylation of tau in the brains was analyzed by Western blots developed with phosphorylation-dependent and site-specific tau antibodies. We found that tau protein in mouse brains was dephosphorylated site-specifically during postmortem (Fig. 1A). Tau sites threonine (Thr) 212 and serine (Ser) 262 were dephosphorylated most rapidly. Tau phosphorylation at these two sites was almost undetectable within 2 min postmortem interval (PMI) (Fig. 1). Phosphorylation of tau at Thr205, Ser214, and Ser396 was also reduced significantly in 2 to 10 min after death (Fig. 1). However, the dephosphorylation at Ser199, Ser404 and Ser422 progressed more slowly (Fig. 1A), in which the phosphorylation levels did not alter or slightly decreased up to 10 min PMI (Fig. 1B). These data suggest that tau is rapidly dephosphorylated during postmortem at multiple phosphorylation sites in a site-specific manner.

### Rapid alteration of phosphorylation of a wide range of proteins in the mouse brain during postmortem

**Figure 2:**
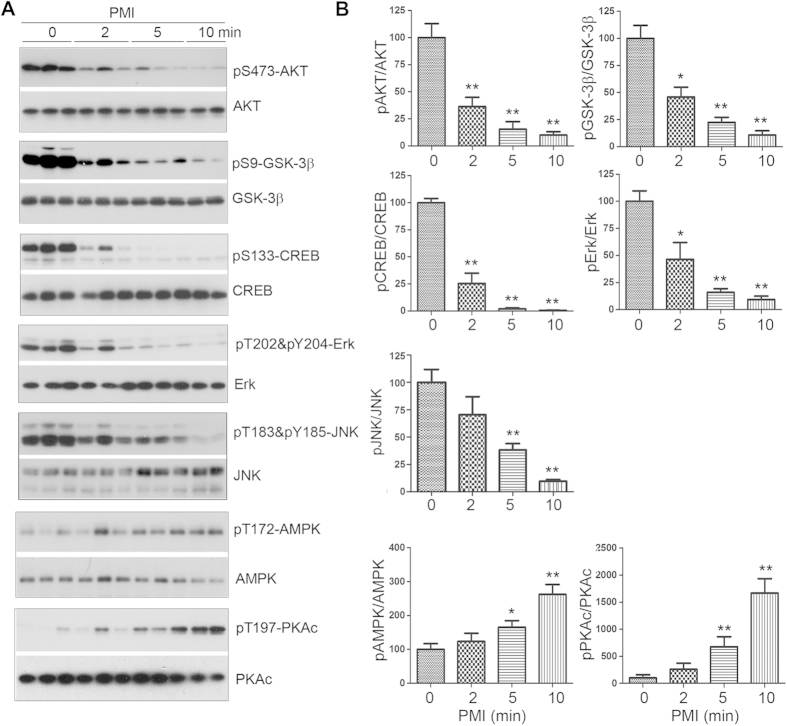
Phosphorylation of proteins in mouse brains is altered during post-mortem. Phosphorylation of proteins in mouse brains with the indicated PMI was analyzed by Western blots developed with phosphorylation dependent antibodies toward the specific protein (**A**). The levels of protein phosphorylation were quantified after being normalized with the corresponding protein and are presented as mean ± SEM (n = 3–4) (**B**). *p < 0.05; **p < 0.01.

To investigate whether the rapid dephosphorylation during postmortem delay is restricted to tau or is a general phenomenon, we determined phosphorylation level of other commonly studied proteins by Western blots. We found that *v*-Akt murine thymoma viral oncogene homolog (AKT) at Ser473, glycogen synthase kinase 3β (GSK-3β) at Ser9, cAMP response element-binding protein (CREB) at Ser133, extracellular signal-regulated kinase (Erk) at Thr202/Tyr204, and c-Jun N-terminal kinase (JNK) at Thr183/Tyr185 in mouse brains all were dephosphorylated very rapidly (Fig. 2). In contrast, the phosphorylation levels of AMP-activated protein kinase α (AMPKα) at Thr172 and protein kinase A (cAMP-dependent protein kinase) catalytic subunit at Thr197 were increased in the mouse brains with postmortem delay up to 10 min (Fig. 2). Collectively, these results suggest that phosphorylation of proteins in mouse brain alters rapidly and dramatically during postmortem and that this alteration is different depending on the specific proteins and phosphorylation sites.

### Dynamic of protein dephosphorylation in the mouse brain during postmortem

**Figure 3:**
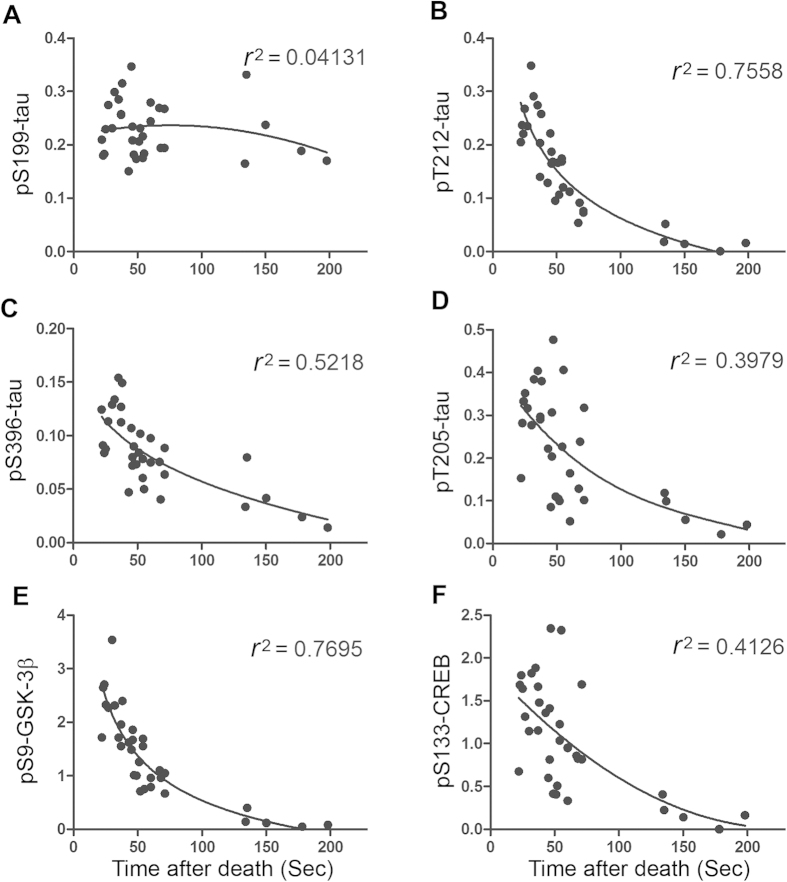
Dynamics of rapid protein dephosphorylation in mouse brains after death. Mice were sacrificed by cervical dislocation and forebrains were dissected out. The times spent between the cervical dislocation and the brains put into dry ice were recorded. The phosphorylation levels of proteins determined by Western blots were plotted against brain collecting times. The non-linear regression was performed, and the coefficient of determination (r^2^) was calculated with Graphpad prism 5.

Because several of the above studied proteins were dephosphorylated more than half of the phosphorylation level within 2 min of postmortem delay, we studied the rapid dynamics of protein dephosphorylation in the mouse brain up to 200 sec after death. We recorded tissue collecting time for each mouse and analyzed phosphorylation levels of proteins in the brains by using Western blots. We then plotted the phosphorylation level against time after death, and the non-linear regression was performed. We found that phosphorylation of tau at Ser199 did not alter significantly up to 200 Sec after death (Fig. 3A), which conforms above result that Ser199 was not sensitive to PMI. However, the phosphorylations of tau at Thr212 and GSK-3β at Ser9 were decreased rapidly. The time to reach 50% reduction was ~40 sec after cervical dislocation. Their coefficient of determination (r^2^) against time after death are 0.7558 and 0.7695, respectively (Fig. 3B,E), indicating that very rapid dephosphorylation is time-dependent. Compared with these two PMI-sensitive sites, the phosphorylation of tau at Thr205, Ser396 and CREB at Ser133 were also reduced, but with a little lower rate (Fig. 3C,D,F). Their 50% reduction time were 60 sec for tau phosphorylated at Thr205 and phosphorylated CREB at Ser133, and 75 sec for tau phosphorylated at Ser396. These results demonstrates the dynamics of rapid dephosphorylation of proteins during tissues processing.

### Rapid alteration of protein phosphorylation in the mouse heart, liver and kidney during postmortem

**Figure 4:**
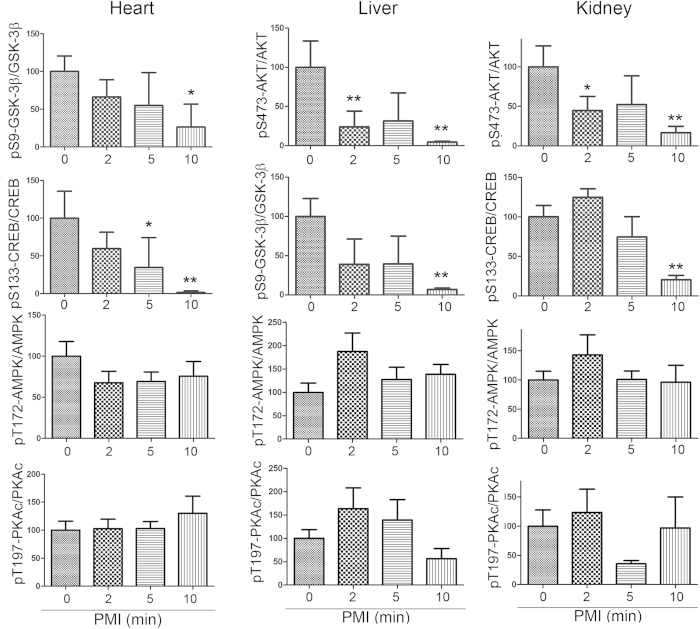
Changes of protein phosphorylation in mouse heart, liver and kidney during postmortem. The heart, liver, and kidney were collected from mice with various PMI. Phosphorylation of proteins in these tissues was analyzed by Western blots developed with phosphorylation dependent antibodies. The phosphorylation level was normalized with the corresponding protein and are presented as mean ± SEM (n = 3–4). *p < 0.05; **p < 0.01.

To learn whether the rapid alteration of protein phosphorylation during postmortem is brain specific, we measured phosphorylation levels of GSK-3β, CREB, AMPK and PKAc in mouse heart, kidney and liver by Western blots. Similar to the brain, we also found that phosphorylation of GSK-3β and CREB in heart, liver and kidney were significantly decreased after death except AKT pSer 473 in liver and kidney and only 10 min PMI in heart and pCREB during 10 min in heart, liver and kidney (Fig. 4). However, the phosphorylation of AMPK and PKAc did not alter significantly in those tissues after death (Fig. 4). Thus, changes of proteins phosphorylation are not only protein- and site-specific, but also tissue dependent.

### Effect of perfusion on phosphorylation of mouse brain proteins

**Figure 5:**
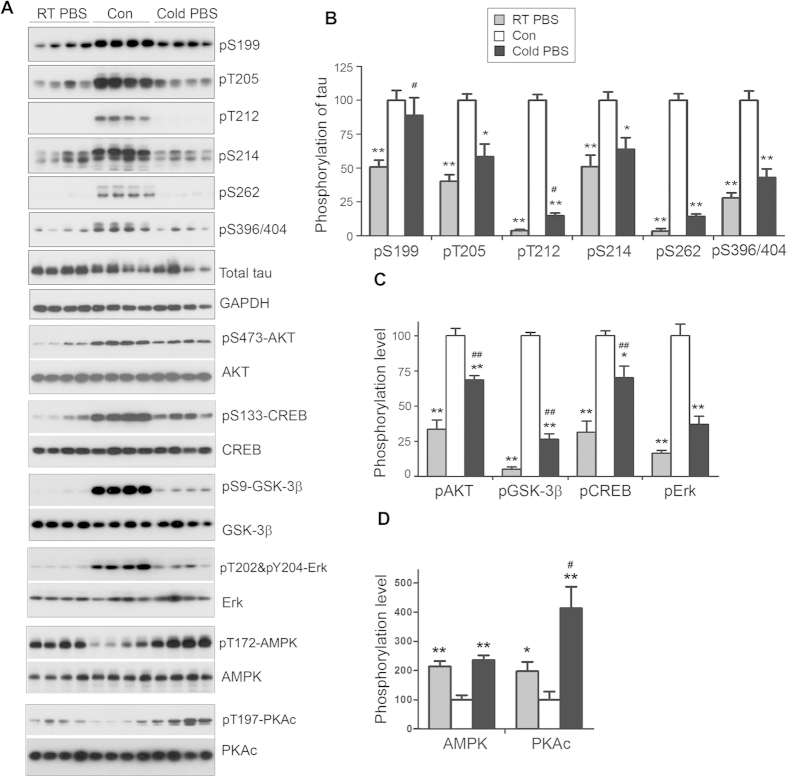
Perfusion causes dramatic changes of protein phosphorylation in the mouse brain. Mice were transcardially perfused with RT-PBS or cold PBS after sacrificed by cervical dislocation. The forebrains were collected and used for protein phosphorylation analysis by Western blots (**A**). The phosphorylation level was quantified after being normalized with the corresponding protein and presented as mean ± SEM (n = 4) (**B**). *^,#^*p* < 0.05; **^,##^*p* < 0.01; *perfusion vs control; ^#^cold PBS vs RT-PBS.

Perfusion of animal with PBS is a regular procedure for immunohistochemical studies. To learn the effect of perfusion on phosphorylation of proteins, we perfused mice through heart after cervical dislocation with either cold or room-temperature PBS (RT-PBS) for 2 min, and then the brains were analyzed for protein phosphorylation by Western blots. For control, the mice brains without perfusion were collected and stored at −80 °C immediately after cervical dislocation. We found that perfusion with either RT-PBS or cold PBS all caused dephosphorylation of brain proteins including tau at Thr205, Thr212, Ser214, Ser262, Ser396 and AKT at Ser473, GSK-3β at Ser9, CREB at Ser133, and Erk at Thr202/Tyr204 (Fig. 5A–C), but the phosphorylation levels of AMPK at Thr172 and PKAc at Thr197 were increased (Fig. 5A, D). However, dephosphorylation of these proteins caused by cold-PBS perfusion was slightly less than by RT-PBS perfusion (Fig. 5A–C).

### Prevention of the alteration of protein phosphorylation during postmortem

**Figure 6:**
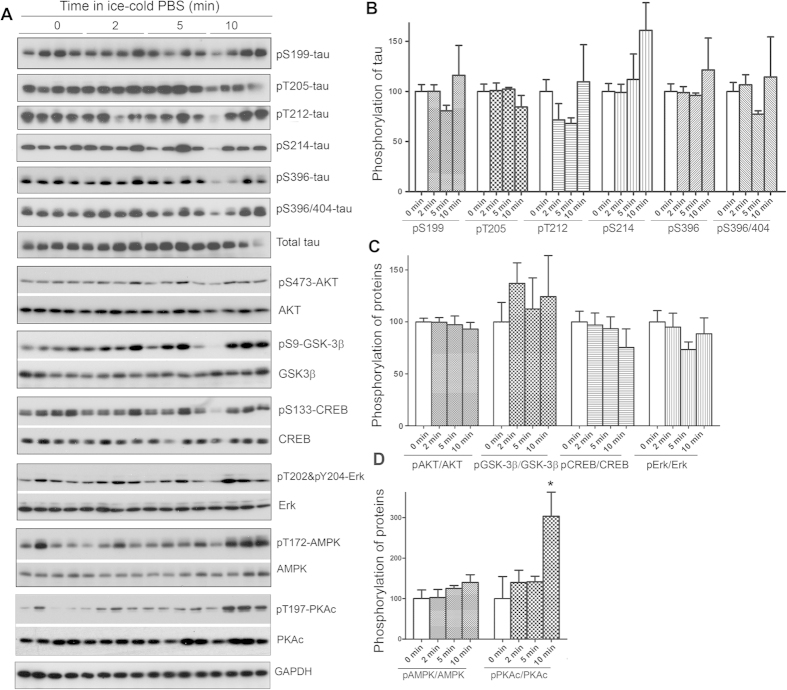
Keeping tissues in ice-cold buffer prevents the alteration of protein phosphorylation in mouse brain. Mice were sacrificed by dislocation. The fore brains were transferred immediately into ice-cold PBS (0 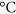) and kept in the ice-cold PBS for various time points. The protein phosphorylation was analyzed by Western blots (**A**). The phosphorylation levels of proteins were normalized by corresponding proteins and are presented as mean ± SEM (n = 4–5) (**B)**. *p < 0.05.

To search for a practical method of preventing or minimizing the alteration of protein phosphorylation during postmortem, we immediately transferred the brains into ice-cold (0 °C) phosphate-buffered saline (PBS) and to cool down them for different time periods after cervical dislocation, and then measured the levels of phosphorylation of different proteins. We found that in general, keeping the brain in ice-cold PBS significantly prevented alteration of protein phosphorylation during postmortem (Fig. 6A–C). The phosphorylation of AMPK and PKAc did not change until 5 min PMI, but the phosphorylation of PKAc was increased in the brain with 10 min PMI (Fig. 6A,D). These data suggest that cooling down brain in ice-cold PBS for 5 min can prevent the changes of protein phosphorylation.

## Discussion

Phosphorylation is the most important post-translational modification of a protein, which regulates its biological activity, subcellular localization and interaction with other biological molecules. In the present study, we studied the progress of alteration of protein phosphorylation in brain, liver, heart and kidney after mouse’s death. We found that the changes of protein phosphorylation appear protein- and/or site-specific manner. Among phosphorylation of proteins detected in present study, we found that most of proteins were dephosphorylated tissue-independently during PMI, but to different extents. In addition to dephosphorylation of proteins after death, we also found phosphorylation levels of AMPK and PKAc was increased up to 10 min PMI in mouse brains. The dephosphorylation of tau at Thr212 and of GSK-3β at Ser9 was very rapid in mouse brain during PMI. Phosphorylation levels of these proteins were reduced by 50% within 40 sec after cervical dislocation, which is regular time period for the brain collection from dead animal. Perfusion with cold PBS for 2 min failed to prevent these rapid dephosphorylation, even though the dephosphorylation in the cold-PBS perfused mouse brain was slowed down a little. Maintaining brain tissues in ice-cold PBS effectively prevented the alteration of protein phosphorylation. The phosphorylation levels of proteins in the mouse brains preserved in ice-cold PBS up to 5 min did not change significantly.

Protein phosphorylation has been implicated in both physiological and pathological events. Postmortem tissues are wildly utilized in the biomedical studies. The alteration of protein phosphorylation during postmortem have be reported^10^,^11^,^12^,^13^,^14^, but it was not received great attention as it should be. In the present study, we investigated the changes of protein phosphorylation after death from 20 sec to 10 min. Though our data are consistent with previous studies in general, surprisingly, we found a rapid alteration of protein phosphorylation in mouse tissues during tissue colleting and perfusion.

The phosphorylation level of proteins *in vivo* is balanced by activities of their kinase(s) and phosphatase(s). Most proteins/sites were found to undergo dephosphorylation in mouse brain after death, suggesting the phosphatases work more effectively than kinases due to cessation of synthesis of ATP in dead brain. Tau is phosphorylated at multiple sites. Consistent with the previous studies^12^,^15^,^16^,^17^, we found here that phosphorylation of tau at most sites is unstable during very short postmortem counted in sec and min with different degree. The phosphorylation of tau at Thr212 Thr205, and Ser262 in mouse brains was reduced to 50% in 40 to 60 sec after cervical dislocation. Several phosphatases are involved in the regulation of tau phosphorylation site-specifically^18^. Protein phosphatase 2 A (PP2A) is the major phosphatase in human brain and occupies 70% of tau phosphatase activity^19^. Inhibition of PP2A significantly increases tau phosphorylation^20^. Thr212, Thr205, Ser262 are dephosphorylated by PP2A most effectively^19^, but phosphorylated by different kinases, which suggests that PP2A, rather than kinases, plays the major contribution in determining the level of tau phosphorylation after death. Ser199 is the least favorite of tau sites of PP2A^19^. A non-PMI-sensitive site status of Ser199 also supports the critical role of PP2A in tau dephosphorylation in dead mouse brain during postmortem.

In addition to tau, as a major protein phosphatase, PP2A dephosphorylates a lot of other proteins *in vitro* and *in vivo*, including AKT, GSK-3β, CREB and Erk tissue-independently^20^,^21^,^22^,^23^,^24^,^25^. These proteins are phosphorylated by various kinases. Their rapid dephosphorylation suggests that PP2A is the major phosphatase in dephosphorylation of these proteins in mouse brains after death. Moreover, Protein phosphatase 1 (PP1) is believed to be the major phosphatase to dephosphorylate CREB^26^,^27^, which is also dephosphorylated after death. Therefore, in addition to PP2A, PP1 may also be involved in the dephosphorylation of proteins in postmortem tissues.

In contrast to dephosphorylation seen in most proteins, we found that phosphorylation of AMPK and PKAc was increased in mouse brain with up to 10 min PMI, which is unexpected. It is possible that the phosphorylation antibodies recognize certain conformation, rather than real-phosphorylation. Several kinases are reported to phosphorylate AMPKα at Thr172, including Ca^2+^/calmodulin-dependent protein kinase kinase-β^28^ and AMPK Kinase, a complex of three proteins, STE-related adaptor (STRAD), mouse protein 25 (MO25), and LKB1 (liver kinase B1)^29^,^30^. AMPK activity is controlled by the ratio of AMP/ATP. When the level of AMP increases, binding of AMP changes the conformation of AMPK and exposes Thr172 for phosphorylation by AMPKK, where binding of AMP renders activated AMPK that is phosphorylated at Thr172 a worse substrate for PP2C^31^,^32^. Thus, increased AMP in the brain for some time after death may enhance the phosphorylation of AMPK and protect its dephosphorylation by PP2C and PP2A, leading to increase in phosphorylation. PKA is phosphorylated by itself or PDK (phosphoinositide-dependent kinase). Increased phosphorylation of PKA in dead mouse brains could be due to auto-activation and/or resistant to the dephosphorylation, which, however, remains to be investigated.

Perfusion with room temperature-PBS is a regular procedure for the immunohistochemical studies. In present study, we found that perfusion caused significant alteration in phosphorylation of proteins. Perfusion with cold-PBS slowed down the rate of alteration of protein phosphorylation a little, but still the alteration of protein phosphorylation was dramatic. These data strongly recommended suggest that perfusion is not recommended for studies on phosphorylation of proteins.

Many enzymes are regulated by phosphorylation, it has been found that the activities of certain enzymes are PMI sensitive^33^,^34^,^35^,^36^. Enzyme activity is affected by temperature significantly. It was reported that focused microwave irradiation of the brain preserves *in vivo* protein phosphorylation^37^, which may kill proteins’ biological activity. Thus, these tissues may not be suitable for the measurement of enzyme activity. In the present study, we found that a rapid transfer of brain tissues from sacrificed animal to ice-cold PBS significantly prevented the alteration in phosphorylation levels of all proteins studied, indicating that ice-cold PBS significantly probably inhibits both phosphatase and kinase activities. Thus, to avoid the alteration of protein phosphorylation after death, it is recommended to cool down the tissues in ice-cold PBS containing a cocktail of phosphatase inhibitors for 5 min and then to dissect the brain tissues.

In summary, protein phosphorylation is progressively altered in protein-, site- and tissue specific manner during postmortem. Some proteins or a protein at some sites are rapidly dephosphorylated during tissue processing, which suggests the tissues should be collected from dead bodies as soon as possible. Perfusion causes dramatic changes on phosphorylation status of many proteins. Cooling down the tissues with ice-cold PBS effectively prevents the alteration of protein phosphorylation in dead tissues.

## Materials and Methods

### Antibodies and Reagents

Primary antibodies used in this study are listed in Table 1. Peroxidase-conjugated anti-mouse and anti-rabbit IgG were obtained from Jackson ImmunoResearch Laboratories (West Grove, PA, USA). The enhanced chemiluminescence (ECL) kit was from Pierce (Rockford, IL, USA). Other chemicals were from Sigma (St. Louis, MO, USA).

### Animals

C57BL/6 and FVB mice were purchased from Charles River Laboratories and housed (4 ~ 5 animals per cage) with a 12/12 h light/dark cycle and with ad libitum access to food and water. The housing, breeding, and animal experiments were in accordance with the approved protocol from our Institutional Animal Care and Use Committee, according to the PHS Policy on Human Care and Use of Laboratory animals.

Adult mice of both genders (~6 months old) were sacrificed by cervical dislocation, and the brains were removed immediately according to each experiment. Animals were then either transcardial perfused (see below) or their forebrains were dissected out in ice cold PBS and frozen in dry ice, and stored at −80 °C for biochemical analyses.

### Transcardial perfusion

C57BL/6 mice were sacrificed via cervical dislocation, and immediately dissected for transcardial perfusion with 25 ml ice cold PBS or RT-PBS, followed by brain dissection for tissue harvest. All procedures were completed as quickly and proficiently as possible. The time consumed for exposure of heart, perfusion and dissection of the brain was ~60, ~100 and ~60 sec, respectively. Totally, the procedures from cervical dislocation to brain tissue harvest were completed in ~4 min.

### Western blot analysis

Mouse brain, heart, liver and kidney tissues were homogenized in pre-chilled buffer containing 50 mM tris-HCl, pH 7.4, 2.0 mM EDTA, 2 mM Na_3_VO_4_, 50 mM NaF, 1 mM 4-(2-aminoethyl) benzenesulfonyl fluoride hydrochloride (AEBSF), 10 μg/ml aprotinin, 10 μg/ml leupeptin, and 10 μg/ml pepstatin A. The homogenates were mixed with 2 × Lemaeli buffer followed by boiling for 5 min. The protein concentration was determined by using A660 kit (Pierce Rockford, IL) according to manufactories manual. The samples were resolved in 10% SDS-PAGE and electro-transferred onto Immobilon-P membrane (Millipore, Bedford, MA, USA). The blots were then probed with primary antibody and developed with the corresponding horseradish peroxidase–conjugated secondary antibody and ECL kit (Pierce, Rockford, IL). Densitometric quantification of protein bands in Western blots was analyzed by using the Multi Gauge V3.0 software (Fuji Photo Film Co., Ltd).

### Statistical analysis

Data were analyzed by one-way ANOVA followed by Tukey’s post hoc tests or unpaired two-tailed *t* tests, using Graphpad. All data are presented as means ± SEM, and *p* < 0.05 was considered statistically significant.

## Additional Information

**How to cite this article**: Wang, Y. *et al.* Rapid alteration of protein phosphorylation during postmortem: implication in the study of protein phosphorylation. *Sci. Rep.* **5**, 15709; doi: 10.1038/srep15709 (2015).

## Figures and Tables

**Table 1:** Primary antibodies employed in this study.

Antibody	Type	Species	Specificity	Modification site	Reference/Source (catalog and/or clone number)
Anti-pS199-tau	Poly-	R	p-tau	Ser199	Invitrogen (44734G)
Anti-pT205-tau	Poly-	R	p-tau	Thr205	Invitrogen (44738G)
Anti-pT212-tau	Poly-	R	p-tau	Thr212	Invitrogen (44740G)
Anti-pS214-tau	Poly-	R	p-tau	Ser214	Invitrogen (44742G)
12E8	Mono-	M	p-tau	Ser262/356	Dr. D. Schenk
Anti-pS396-tau	Poly-	R	p-tau	Ser396	Invitrogen (44752G)
Anti-pS404-tau	Poly-	R	p-tau	Ser404	Invitrogen (44758G)
PHF-1	Mono-	M	p-tau	Ser396/404	Dr. P. Davies
R145d	Poly-	R	p-tau	Ser422	Pei *et al.*,^38^
R134d	Poly-	R	Tau		Pei *et al.*,^38^
Anti-AKT	Mono-	R	AKT		Cell Signaling (4685/11E7)
Anti-pSer473-AKT	Poly-	R	p-AKT	Ser473	Cell Signaling (9271)
Anti-pSer9-GSK-3β	Mono-	R	p-GSK-3β	Ser9	Cell Signaling (9323/5B3)
Anti-GSK-3β	Mono-	R	GSK-3β		Cell Signaling (9315/27C10)
Anti-pS133-CREB	Mono-	R	p-CREB	Ser133	Cell Signaling (9198/87G3)
Anti-CREB	Mono-	R	CREB		Cell Signaling (9197/48H2)
Anti-ERK1/2	Poly-	R	ERK1/2		Cell Signaling (9102)
Anti-pT202/Y204-ERK1/2	Poly-	R	p-ERK1/2	Thr202/Tyr204	Cell Signaling (9101)
Anti-JNK	Mono-	R	JNK		Cell Signaling (56G8)
Anti-pT183/Y185-JNK	Poly	R	p-JNK	Thr183/Tyr204	Cell Signaling (9251)
Anti-pT197-PKAc	Mono-	R	p-PKAc	Thr197	Cell Signaling (5665/D45D3)
Anti-PKAc*α*&β	Poly-	R	PKAcα&β		Santa-Cruz (sc-903&sc-904)
Anti-pT172-APMP*α*	Mono-	R	pAMPK	pThr172	Cell Signaling (2535/40H9)
Anti-AMPKα	Poly-	R	AMPK		Cell Signaling (2532)
Anti-GAPDH	Poly-	R	GAPDH		Santa-Cruz (sc-25778)

AMPKa, 5′ AMP-activated protein kinase a subunit; AKT, *v*-Akt murine thymoma viral oncogene homolog; CREB, cAMP response element-binding protein; Erk, extracellular signal-regulated kinases; GAPDH, glyceraldehyde-3-phosphate dehydrogenase; GSK-3β, glycogen synthase kinase-3β; PKAc, cAMP-dependent protein kinase catalytic subunit; Mono-, monoclonal; p-, phosphorylated; Poly-, polyclonal; M, Mouse; R, Rabbit; Ser, serine; Thr, threonine; and Tyr, tyrosine.

## References

[b1] Levene P. A. & Beatty W. A. On the phosphotungstates of certain aminoacids. J Exp Med 8, 463–466 (1906). 19867052 10.1084/jem.8.3.463 PMC2124627

[b2] Fischer E. H. & Krebs E. G. Conversion of phosphorylase b to phosphorylase a in muscle extracts. J Biol Chem 216, 121–132 (1955). 13252012

[b3] Cohen P. The origins of protein phosphorylation. Nat Cell Biol 4, E127–130 (2002). 11988757 10.1038/ncb0502-e127

[b4] Johnson L. N. The regulation of protein phosphorylation. Biochem Soc Trans 37, 627–641 (2009). 19614568 10.1042/BST0370627

[b5] Cohen P. The regulation of protein function by multisite phosphorylation—a 25 year update. Trends Biochem Sci 25, 596–601 (2000). 11116185 10.1016/s0968-0004(00)01712-6

[b6] Iqbal K. *et al.* Defective brain microtubule assembly in Alzheimer’s disease. Lancet 2, 421–426 (1986). 2874414 10.1016/s0140-6736(86)92134-3

[b7] Grundke-Iqbal I. *et al.* Abnormal phosphorylation of the microtubule-associated protein tau (tau) in Alzheimer cytoskeletal pathology. Proc Natl Acad Sci USA 83, 4913–4917 (1986). 3088567 10.1073/pnas.83.13.4913 PMC323854

[b8] Manning G., Whyte D. B., Martinez R., Hunter T. & Sudarsanam S. The protein kinase complement of the human genome. Science 298, 1912–1934 (2002). 12471243 10.1126/science.1075762

[b9] Wang J. Z. & Liu F. Microtubule-associated protein tau in development, degeneration and protection of neurons. Prog Neurobiol 85, 148–175 (2008). 18448228 10.1016/j.pneurobio.2008.03.002

[b10] Li J., Gould T. D., Yuan P., Manji H. K. & Chen G. Post-mortem interval effects on the phosphorylation of signaling proteins. Neuropsychopharmacol 28, 1017–1025 (2003). 10.1038/sj.npp.1300112 12637955

[b11] Scharf M. T., Mackiewicz M., Naidoo N., O’Callaghan J. P. & Pack A. I. AMP-activated protein kinase phosphorylation in brain is dependent on method of killing and tissue preparation. J Neurochem 105, 833–841 (2008). 18088373 10.1111/j.1471-4159.2007.05182.x PMC3778452

[b12] Gartner U., Janke C., Holzer M., Vanmechelen E. & Arendt T. Postmortem changes in the phosphorylation state of tau-protein in the rat brain. Neurobiol Aging 19, 535–543 (1998). 10192212 10.1016/s0197-4580(98)00094-3

[b13] Li X., Friedman A. B., Roh M. S. & Jope R. S. Anesthesia and post-mortem interval profoundly influence the regulatory serine phosphorylation of glycogen synthase kinase-3 in mouse brain. J Neurochem 92, 701–704 (2005). 15659239 10.1111/j.1471-4159.2004.02898.x PMC1850892

[b14] Oka T., Tagawa K., Ito H. & Okazawa H. Dynamic changes of the phosphoproteome in postmortem mouse brains. PLoS One 6, e21405 (2011). 21731734 10.1371/journal.pone.0021405 PMC3120861

[b15] Burack M. A. & Halpain S. Site-specific regulation of Alzheimer-like tau phosphorylation in living neurons. Neuroscience 72, 167–184 (1996). 8730715 10.1016/0306-4522(95)00546-3

[b16] Matsuo E. S. *et al.* Biopsy-derived adult human brain tau is phosphorylated at many of the same sites as Alzheimer’s disease paired helical filament tau. Neuron 13, 989–1002 (1994). 7946342 10.1016/0896-6273(94)90264-x

[b17] Song J. *et al.* Low initial tau phosphorylation in human brain biopsy samples. Neurobiol Aging 18, 475–481 (1997). 9390773 10.1016/s0197-4580(97)00043-2

[b18] Liu F., Liang Z. & Gong C. X. Hyperphosphorylation of tau and protein phosphatases in Alzheimer disease. Panminerva Med 48, 97–108 (2006). 16953147

[b19] Liu F., Grundke-Iqbal I., Iqbal K. & Gong C. X. Contributions of protein phosphatases PP1, PP2A, PP2B and PP5 to the regulation of tau phosphorylation. Eur J Neurosci 22, 1942–1950 (2005). 16262633 10.1111/j.1460-9568.2005.04391.x

[b20] Qian W. *et al.* PP2A regulates tau phosphorylation directly and also indirectly via activating GSK-3beta. J Alzheimers Dis 19, 1221–1229 (2010). 20308788 10.3233/JAD-2010-1317

[b21] Zhou B., Wang Z. X., Zhao Y., Brautigan D. L. & Zhang Z. Y. The specificity of extracellular signal-regulated kinase 2 dephosphorylation by protein phosphatases. J Biol Chem 277, 31818–31825 (2002). 12082107 10.1074/jbc.M203969200

[b22] Resjo S. *et al.* Protein phosphatase 2 A is the main phosphatase involved in the regulation of protein kinase B in rat adipocytes. Cell Signal 14, 231–238 (2002). 11812651 10.1016/s0898-6568(01)00238-8

[b23] Wang Y. *et al.* Cross talk between PI3K-AKT-GSK-3beta and PP2A pathways determines tau hyperphosphorylation. Neurobiol Aging 36, 188–200 (2015). 25219467 10.1016/j.neurobiolaging.2014.07.035

[b24] Zakany R. *et al.* Protein phosphatase 2 A is involved in the regulation of protein kinase A signaling pathway during *in vitro* chondrogenesis. Exp Cell Res 275, 1–8 (2002). 11925100 10.1006/excr.2002.5487

[b25] Wadzinski B. E. *et al.* Nuclear protein phosphatase 2 A dephosphorylates protein kinase A-phosphorylated CREB and regulates CREB transcriptional stimulation. Mol Cell Biol 13, 2822–2834 (1993). 8386317 10.1128/mcb.13.5.2822-2834.1993 PMC359667

[b26] Alberts A. S., Montminy M., Shenolikar S. & Feramisco J. R. Expression of a peptide inhibitor of protein phosphatase 1 increases phosphorylation and activity of CREB in NIH 3T3 fibroblasts. Mol Cell Biol 14, 4398–4407 (1994). 7516466 10.1128/mcb.14.7.4398-4407.1994 PMC358811

[b27] Lee B., Butcher G. Q., Hoyt K. R., Impey S. & Obrietan K. Activity-dependent neuroprotection and cAMP response element-binding protein (CREB): kinase coupling, stimulus intensity, and temporal regulation of CREB phosphorylation at serine 133. J Neurosci 25, 1137–1148 (2005). 15689550 10.1523/JNEUROSCI.4288-04.2005 PMC6725976

[b28] Woods A. *et al.* Ca2+/calmodulin-dependent protein kinase kinase-beta acts upstream of AMP-activated protein kinase in mammalian cells. Cell Metab 2, 21–33 (2005). 16054096 10.1016/j.cmet.2005.06.005

[b29] Hawley S. A. *et al.* Characterization of the AMP-activated protein kinase kinase from rat liver and identification of threonine 172 as the major site at which it phosphorylates AMP-activated protein kinase. J Biol Chem 271, 27879–27887 (1996). 8910387 10.1074/jbc.271.44.27879

[b30] Shaw R. J. *et al.* The tumor suppressor LKB1 kinase directly activates AMP-activated kinase and regulates apoptosis in response to energy stress. Proc Natl Acad Sci USA 101, 3329–3335 (2004). 14985505 10.1073/pnas.0308061100 PMC373461

[b31] Suter M. *et al.* Dissecting the role of 5′-AMP for allosteric stimulation, activation, and deactivation of AMP-activated protein kinase. J Biol Chem 281, 32207–32216 (2006). 16943194 10.1074/jbc.M606357200

[b32] Davies S. P., Helps N. R., Cohen P. T. & Hardie D. G. 5′-AMP inhibits dephosphorylation, as well as promoting phosphorylation, of the AMP-activated protein kinase. Studies using bacterially expressed human protein phosphatase-2 C alpha and native bovine protein phosphatase-2AC. FEBS Lett 377, 421–425 (1995). 8549768 10.1016/0014-5793(95)01368-7

[b33] Bowen D. M., Smith C. B., White P. & Davison A. N. Neurotransmitter-related enzymes and indices of hypoxia in senile dementia and other abiotrophies. Brain 99, 459–496 (1976). 11871 10.1093/brain/99.3.459

[b34] Puymirat J. *et al.* Post mortem stability and storage in the cold of brain enzymes. J Neurochem 32, 449–454 (1979). 33233 10.1111/j.1471-4159.1979.tb00370.x

[b35] Roytta M., Laaksonen H., Frey H., Riekkinen P. & Rinne U. K. Critical evaluation of the postmortem factors influencing neurochemical analyses of brain autopsies. Acta Neurol Scand 61, 88–106 (1980). 6104885 10.1111/j.1600-0404.1980.tb01471.x

[b36] Spokes E. G. & Koch D. J. Post-mortem stability of dopamine, glutamate decarboxylase and choline acetyltransferase in the mouse brain under conditions simulating the handling of human autopsy material. J Neurochem 31, 381–383 (1978). 671036 10.1111/j.1471-4159.1978.tb12477.x

[b37] O’Callaghan J. P. & Sriram K. Focused microwave irradiation of the brain preserves *in vivo* protein phosphorylation: comparison with other methods of sacrifice and analysis of multiple phosphoproteins. J Neurosci Methods 135, 159–168 (2004). 15020100 10.1016/j.jneumeth.2003.12.006

[b38] Pei J. J. *et al.* Subcellular distribution of protein phosphatases and abnormally phosphorylated tau in the temporal cortex from Alzheimer’s disease and control brains. J Neural Transm 105, 69–83 (1998). 9588762 10.1007/s007020050039

