# Corrigendum: Rapid alteration of protein phosphorylation during postmortem: implication in the study of protein phosphorylation

**DOI:** 10.1038/srep17241

**Published:** 2016-01-07

**Authors:** Yifan Wang, Yanchong Zhang, Wen Hu, Shutao Xie, Cheng-Xin Gong, Khalid Iqbal, Fei Liu

Scientific Reports 5: Article number: 1570910.1038/srep15709; published online: 10292015; updated: 01072016.

In this Article, Khalid Iqbal is incorrectly affiliated with ‘Jiangsu Key Laboratory of Neuroregeneration, Co-innovation Center of Neuroregeneration, Nantong University, Nantong, Jiangsu 226001, P. R. China.’ The correct affiliation is listed below:

Department of Neurochemistry, Inge Grundke-Iqbal Research Floor, New York State Institute for Basic Research in Developmental Disabilities, Staten Island, New York 10314, USA

